# P2Y12 Antiplatelet Choice for Patients with Chronic Kidney Disease and Acute Coronary Syndrome: A Systematic Review and Meta-Analysis

**DOI:** 10.3390/jpm11030222

**Published:** 2021-03-21

**Authors:** Sohyun Park, Yeo Jin Choi, Ji Eun Kang, Myeong Gyu Kim, Min Jung Geum, So Dam Kim, Sandy Jeong Rhie

**Affiliations:** 1 Division of Life and Pharmaceutical Sciences Graduate School, Ewha Womans University, Seoul 03760, Korea; sohyun.sh.park@gmail.com; 2 Department of Pharmacy, National Medical Center, Seoul 04564, Korea; jjadu@nmc.ac.kr; 3 Graduate School of Clinical Pharmacy, CHA University, Seongnam 13488, Korea; yjchoi@cha.ac.kr; 4 College of Pharmacy, Ewha Womans University, Seoul 03760, Korea; kimmg@ewha.ac.kr (M.G.K.); claire.sodame.kim@gmail.com (S.D.K.); 5 Graduate School of Pharmaceutical Sciences, Ewha Womans University, Seoul 03760, Korea; MJGEUM@yuhs.ac; 6 Department of Pharmacy, Severance Hospital, Yonsei University Health System, Seoul 03722, Korea

**Keywords:** acute coronary syndrome, antiplatelet, dual antiplatelet therapy, P2Y12 inhibitors, chronic kidney disease, hemodialysis, clopidogrel resistance, high on-treatment of platelet reactivity

## Abstract

This study aims to evaluate potentially appropriate antiplatelet therapy in patients with chronic kidney disease. A systematic analysis was conducted to identify the clinical outcomes of available antiplatelet therapy regimens with enhanced platelet inhibition activity (intervention of 5 regimens) over the standard dose of clopidogrel-based dual antiplatelet therapy in patients with renal insufficiency. An electronic keyword search was performed on Pubmed, Embase, and Cochrane Library per PRISMA guidelines. We performed a prespecified net clinical benefit analysis (a composite of the rates of all-cause or cardiac-related death, myocardial infarction, major adverse cardiac outcomes, and minor and major bleeding), and included 12 studies. The intervention substantially lowered the incidence of all-cause mortality (RR 0.67; *p* = 0.003), major adverse cardiac outcomes (RR 0.79; *p* < 0.00001), and myocardial infarction (RR 0.28; *p* = 0.00007) without major bleeding (RR 1.14; *p* = 0.33) in patients with renal insufficiency, but no significant differences were noticed with cardiac-related mortality and stent thrombosis. The subgroup analysis revealed substantially elevated bleeding risk in patients with severe renal insufficiency or on hemodialysis (RR 1.68; *p* = 0.002). Our study confirmed that the intervention considerably enhances clinical outcomes in patients with renal insufficiency, however, a standard dose of clopidogrel-based antiplatelet therapy is favorable in patients with severe renal insufficiency.

## 1. Introduction

The current guidelines published by the American Heart Association in conjunction with the College of Cardiology (AHA/ACC) and the European Society of Cardiology (ESC) highly recommend dual antiplatelet therapy (DAPT) with aspirin and a P2Y12 receptor inhibitor in patients with acute coronary syndromes (ACS) to avoid the increased risk of platelet aggregation associated with ACS progression and prognosis [1,2]. Appropriate use of DAPT offers intense platelet inhibition compared to single antiplatelet therapy, subsequently providing secondary prevention of stent thrombosis and new-onset myocardial infarction (MI) and decreased mortality after a percutaneous coronary intervention (PCI) or ACS. However, DAPT may be associated with elevated bleeding risk [2,3]. The optimal DAPT regimen and duration are tailored to patients’ risk indicators of bleeding and clinical presentation during therapy. The guidelines suggest a 12-month DAPT treatment in patients with a low risk of bleeding and one month in patients with a very high risk of bleeding [2]. Active bleeding disorders, a low body weight, old age, concomitant drug use with potential bleeding risk, reduced estimated glomerular filtration rate (eGFR), and poor cytochrome P450 metabolizers may alter the pharmacokinetic and pharmacodynamic profiles of antiplatelets in the DAPT regimen and can influence clinical outcomes of DAPT [4,5]. Nonetheless, precise estimation of bleeding risk is impracticable in patients with multiple comorbidities, especially those with renal insufficiency [6,7].

Chronic kidney disease (CKD), defined as an eGFR < 60 mL/min per 1.73 m^2^, has several disease-specific features affecting ACS prognosis [8]. Almost 30% of patients diagnosed with ST-elevation myocardial infarction (STEMI) and 40% of patients with non-ST elevation myocardial infarctions (NSTEMI) are diagnosed with CKD. ACS-related mortality is reportedly substantially elevated in relation to renal insufficiency, predisposing patients on dialysis to the most pronounced mortality [5,8,9]. Another concern is that CKD patients are at elevated risk for bleeding regardless of antiplatelet treatment, as indicated by a study that demonstrated a 1.7-fold increased bleeding risk in CKD patients without a history of stroke, MI, or peripheral arterial disease [10]. The 2020 ESC guidelines identified CKD as a risk factor for bleeding after PCI in patients with ACS, and the Academic Research Consortium for High Bleeding Risk (ARC-HBR) classified severe or end-stage CKD (eGFR < 30 mL/min) as a major risk factor for bleeding and moderate CKD (eGFR 30–59 mL/min) as a minor risk factor for bleeding [2,11]. However, dose adjustments based on the degree of renal insufficiency are not required for a DAPT regimen in patients with ACS and CKD considering the elevated thrombotic risks. Moreover, a suitable antiplatelet regimen in these patient populations remains unclear.

The current ESC guideline recommends the use of potent P2Y12 inhibitors, ticagrelor or prasugrel, over clopidogrel, which has response variability associated with genetic mutation and drug interactions. However, the clear recommendation regarding selection of P2Y12 inhibitors in CKD patients including those with severe CKD (eGFR < 15 mL/min) is unavailable [2,9,12,13]. Another interesting aspect of clopidogrel is associated with variable platelet reactivity in relation to the degree of renal insufficiency; platelet reactivity increases with decreased renal function, which subsequently increases the risk of major adverse cardiovascular events (MACEs), ischemic events, and hospitalization in patients with CKD regardless of appropriate standard doses of clopidogrel-based DAPT dose and duration [14]. Recent studies have demonstrated improved clinical outcomes, such as MACE, with other potent oral P2Y12 receptor inhibitors over clopidogrel in ACS patients. However, the risks and benefits remain unclear as these agents significantly increase bleeding, which those with CKD are already susceptible to [10,15,16]. Besides, the efficacy and safety of DAPT with potent P2Y12 inhibitors in patients with renal sufficiency including patients on dialysis who have the most pronounced ACS-related mortality and bleeding risk are not fully determined because of limited numbers of clinical trials in these populations [14,15,16]. As a substantial portion of the population diagnosed with ACS has CKD, and these patients may have thrombotic events and an elevated risk for bleeding, appropriate antiplatelet treatment cannot be neglected in this population. Therefore, the aim of this study is to investigate potentially appropriate antiplatelet therapy in patients with CKD including those on dialysis by evaluating the safety and efficacy of different antiplatelet therapy regimens with enhanced antiplatelet activity to overcome clopidogrel response variability [13], over the standard clopidogrel-based DAPT regimen.

## 2. Materials and Methods

### 2.1. Data Sources and Search Strategy

We searched PubMed, Embase, and Cochrane Central Register of Controlled Trials (CENTRAL), without year and language restrictions. The initial database search involved a combination of keywords and Medical Subject Headings (MeSH) of ‘renal,’ ‘renal dialysis,’ ‘renal replacement therapy,’ ‘chronic renal disease,’ ‘chronic kidney failure,’ ‘renal insufficiency,’ ‘clopidogrel,’ ‘prasugrel,’ ‘ticagrelor,’ ‘P2Y12 inhibitor,’ ‘cilostazol,’ and ‘antiplatelet’ in the title/abstract. Search filters were set as ‘clinical trials,’ ‘randomized controlled trials,’ and ‘humans’. The last search was updated in October of 2020. We also manually searched the references of eligible review articles to identify additional research for analysis. Trials were potentially eligible regardless of the study phase, dose schedule, and study region. We followed the Preferred Reporting Items for Systematic Reviews and Meta-analyses (PRISMA) guidelines regarding the search strategy and selection process [17]. 

### 2.2. Study Selection and Data Extraction

Two reviewers (Park and Choi) independently screened the titles and abstracts of all studies that were identified from the literature search to verify eligibility. Disagreements related to the study selection were further discussed until a consensus was reached. Any disagreements not meeting consensus were resolved by a third researcher. 

From each eligible clinical trial, we extracted the study and article characteristics (including the name of the first author, year of publication, study design, study period, and study region), study population (including the number of randomly assigned patients, the types of concomitant cardiovascular disease, and the status of renal impairment), study intervention (including comparator, drug names and dosage, schedule, and intervention duration), outcomes related to efficacy (including mortality, MACE, MI, and stent thrombosis) and safety (including major and minor bleeding), and intermediate measurements of platelet function (including inhibition of platelet aggregation (IPA) and platelet reactivity unit (PRU)). Duplicated studies, review articles, commentaries, editorials, conference abstracts, case reports, and protocols were excluded. The PICOS (Patients, Intervention, Comparator, Outcomes, and Study design) summary of our study is shown in Table 1 [18].

### 2.3. End Points

The efficacy outcomes of interest were all-cause mortality, cardiac-related mortality, MACE, MI, and stent thrombosis. The safety outcomes were major and minor bleeding events and the intermediate outcomes were IPA and PRU.

### 2.4. Assessment of Bias Risk and Evidence

Two independent reviewers (Park and Choi) assessed the methodological quality of randomized controlled trials (RCTs) based on Cochrane’s Risk of Bias (RoB) [19]. The studies were scored as low, unclear, or high in the following domains: randomization sequence generation, allocation concealment, blinding of participants and personnel, blinding of outcome assessment, incomplete outcome data, selective reporting, and other potential bias. The Risk of Bias Assessment Tool for Nonrandomized Study (ROBINS-I) was adapted to evaluate the study quality of non-RCTs regarding the selection of participants, confounding variables, measurement interventions, blinding for assessment, incomplete outcome data, and selective outcome reporting [20,21]. Any disagreements on the risk of bias and quality of evidence were resolved by a third reviewer (Rhie). Egger’s test and funnel plots were utilized to detect possible publication bias; a *p*-value >0.05 and a symmetric funnel plot imply a low risk of publication bias. 

### 2.5. Statistical Methods

Dichotomous data for each eligible study, including all-cause mortality, cardiac-related mortality, MACE, MI, stent thrombosis, and major or minor bleeding events, were analyzed with relative risks (RR) and 95% confidence intervals (CI) to estimate the risks in patients with ACS receiving various interventions (doubled loading dose (LD) of clopidogrel-based DAPT, doubled maintenance dose (MD) of clopidogrel-based DAPT, prasugrel-based DAPT, ticagrelor-based DAPTs, and triple antiplatelet therapy with cilostazol) versus a control (standard dose of clopidogrel-based DAPT). We reversely calculated the number of patients in the study when the results were reported as a percentage (%) in the original work.

Heterogeneity across the included studies was first evaluated by Cochran’s Q test (considered significant for *p* < 0.10) [22] and quantified I2 index [23]. The study outcomes with high heterogeneity (I2 > 50%) were analyzed by a random-effect model (Mantel-Haenszel), while those with low heterogeneity (I2 < 50%) were evaluated by a fixed-effect model.

Subgroup analysis was performed to explore the high heterogeneities observed in the bleeding analysis. After the overall bleeding analysis was conducted to compare the interventions and control in patients with CKD, including those on hemodialysis, the risk of major and minor bleeding according to the degree of renal insufficiency was further analyzed via a comparative analysis on bleeding risks between patients with CKD of moderate renal insufficiency (eGFR < 60 mL/min) and those with severe CKD (eGFR < 30 mL/min) or on hemodialysis. *p*-values were estimated by two-sided tests. Any p-values less than 0.05 were considered statistically significant. The outcomes of interest were evaluated by pooling raw data from individual clinical trials. All statistical analyses were performed using Review Manager (RevMan) Version 5. 4 (The Cochrane Collaboration, 2020) [24]. 

## 3. Results

### 3.1. Study Search and Selection

**Figure 1:**
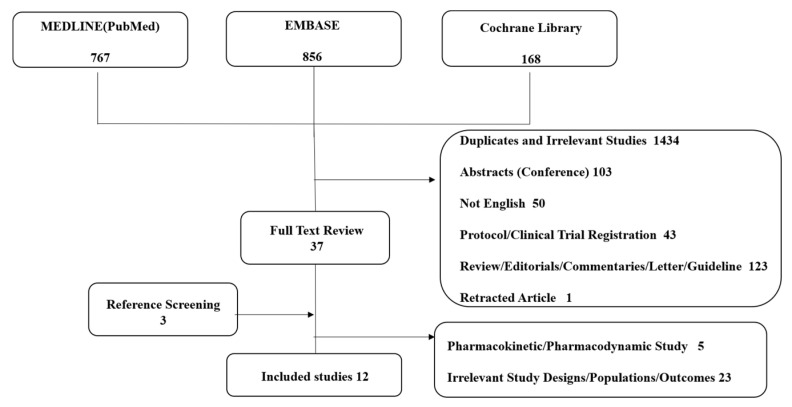
Study selection diagram (Preferred Reporting Items for Systematic Reviews and Meta-analyses (PRISMA)).

The primary literature search from MEDLINE, Embase, and Cochrane yielded 1,791 articles (Figure 1). A total of 37 articles were eligible for full-text review after excluding duplicated or irrelevant studies (1434), abstracts and conference posters (103), study protocols or clinical trial registrations (43), reviews, editorials, commentaries, letters, and guidelines (123), retracted articles (1), and studies not written in English (50). A manual reference screening identified three articles eligible for full-text review. After excluding irrelevant study designs and outcomes (28), a total of 12 clinical trials [5,25,26,27,28,29,30,31,32,33,34,35] were included in this study, including 33,658 patients with CKD on antiplatelet therapy. 

### 3.2. Study Characteristics

Table 2 provides details on the characteristics of included studies. All patients had ACS with CKD, including those on hemodialysis. This study included six randomized trials [5,30,32,33,34,35], two post hoc analyses from previous clinical trials [28,31], and four observational studies [25,26,27,29]. The study regions included China [30], Japan [31], Korea [26,29,32,33,34,35], Sweden [27], and the United States [25], along with two multinational studies [5,28]. Eight studies [5,25,26,27,28,29,30,31] recruited ACS patients with creatinine clearance (CrCl) or eGFR less than 60 mL/min, and four studies [32,33,34,35] recruited patients on hemodialysis. In the original studies, the patients’ characteristics (such as age, ACS status, and level of renal impairment) were well balanced between the arms.

By reviewing the original studies, five different antiplatelet therapies were found to assess the efficacy and safety of this treatment. One was doubled LD clopidogrel-based DAPT [29], three were doubled MD clopidogrel-based DAPT [30,34,35], three were prasugrel-based DAPT [5,25,31], four were ticagrelor-based DAPT [27,28,32,33], and two were triple therapy (a standard dose of clopidogrel-based DAPT with cilostazol, and one was doubled MD clopidogrel-based DAPT) [26,35]. All 12 trials had a control of a standard dose of clopidogrel-based DAPT. Two trials [31,33] demonstrated efficacy with a reduced dosage considering the decreased renal function of one with prasugrel [31] and one with ticagrelor [33].

The intervention outcomes were expressed either in efficacy outcomes, such as mortality, MACE, MI, and stent thrombosis [25,26,27,28,29,30], or intermediate outcomes, such as platelet reactivity [5,31,32,33,34,35]. The safety outcomes were evaluated by the incidence of major and minor bleeding events [25,26,27,28,29,30,32,33,34,35]. The original studies assessing the interventional efficacy in patients on hemodialysis showed intermediate and safety outcomes [32,33,34,35]. The quality assessments of each study are described in Supplementary Tables S1 and S2. The risk of bias was generally acceptable as inferred by Egger’s test (*p* > 0.05) and the symmetric funnel plots (Supplementary Figure S1).

### 3.3. Outcomes in Patients with CKD

**Figure 2:**
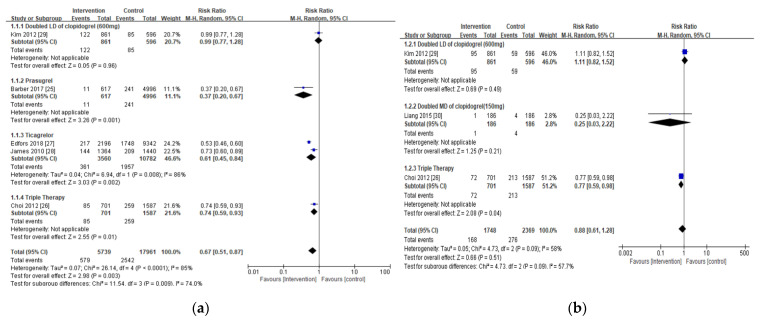
Forest plots of antiplatelet therapy effects on mortality in chronic kidney disease CKD patients: (**a**) all-cause mortality; (**b**) cardiac-related mortality. Blue indicates the risk ratio evaluated from each study and black indicates the overall risk ratio of the antiplatelet regimen.

Compared with the standard dose of clopidogrel-based DAPT (control), the intervention substantially lowered the risk of all-cause mortality in patients with CKD and ACS (RR 0.67; 95% CI 0.51, 0.87; *p* = 0.003), but no significant difference was noted for cardiac-related death (RR 0.88; 95% CI 0.61, 1.28, *p* = 0.51) (Figure 2a,b).

**Figure 3:**
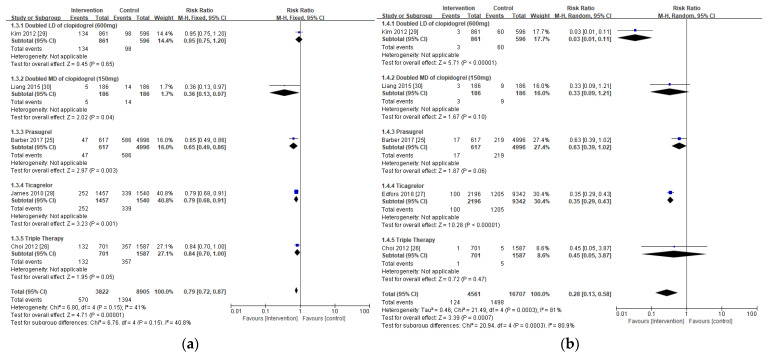

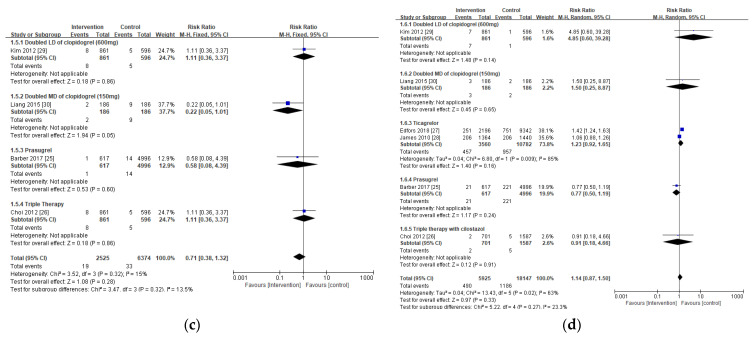
Forest plots of clinical outcomes in CKD patients from antiplatelet therapy: (**a**) major adverse cardiovascular events (MACE); (**b**) myocardial infarction (MI); (**c**) stent thrombosis; and (**d**) major bleeding risks. Blue indicates the risk ratio evaluated from each study and black indicates the overall risk ratio of the antiplatelet regimen.

Furthermore, the intervention considerably decreased the risk of MACE (RR 0.79; 95% CI 0.72, 0.87; *p* < 0.00001, Figure 3a) and MI (RR 0.28; 95% CI 0.13, 0.58; *p* = 0.00007, Figure 3b) in subjects with CKD compared to the controls without increased risk for major bleeding (RR 1.14; 95% CI 0.87, 1.50; *p* = 0.33, Figure 3d). There was no significant difference in the risk of stent thrombosis between the intervention and the control (RR 0.71; 95% CI 0.38, 1.32; *p* = 0.28, Figure 3c).

### 3.4. Safety Outcomes in Patients with CKD, Including Those on Hemodialysis

**Figure 4:**
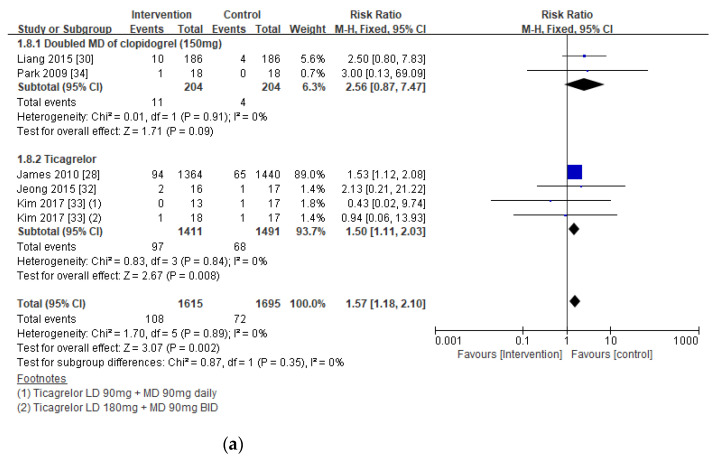

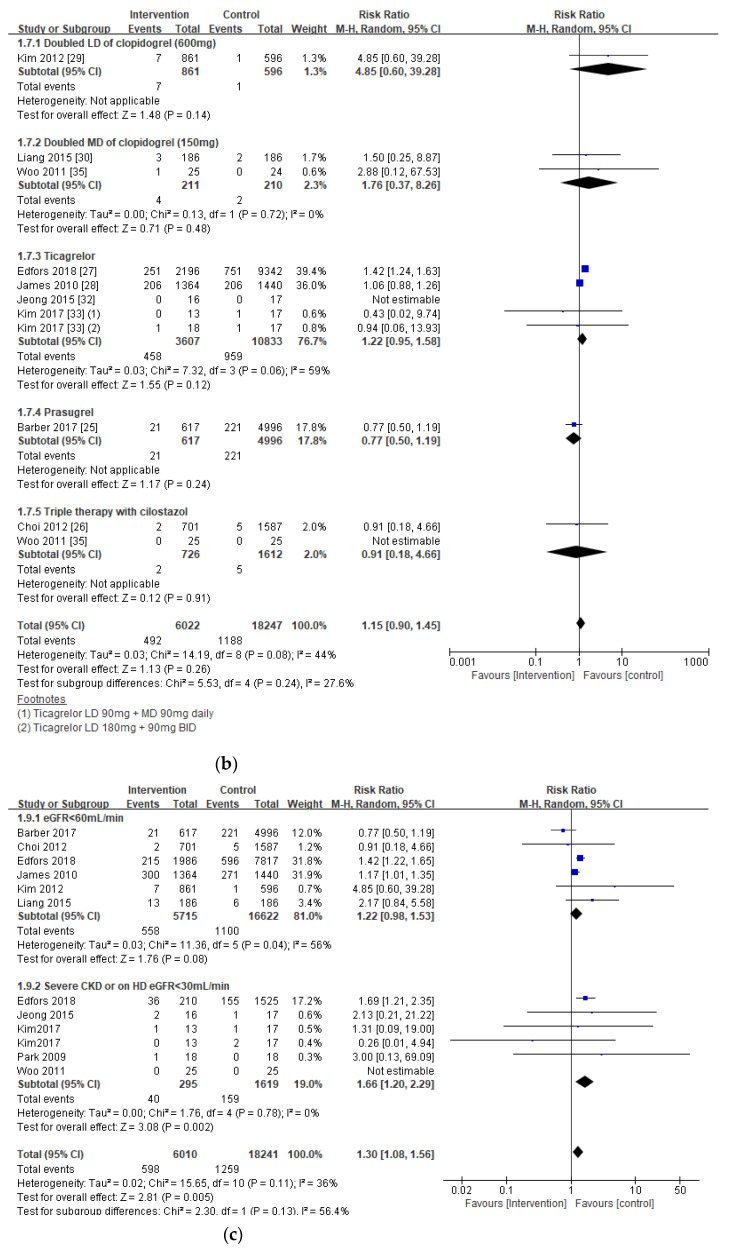
Forest plots of bleeding risks associated with antiplatelet therapy in all CKD patients, including those on hemodialysis: (**a**) major bleeding; (**b**) minor bleeding; and (**c**) subgroup analysis of major and minor bleeding between estimated glomerular filtration rate (eGFR) < 60 mL/min and eGFR < 30 mL/min or hemodialysis (HD). Blue indicates the risk ratio evaluated from each study and black indicates the overall risk ratio of the antiplatelet regimen.

The risk of major bleeding events in patients with CKD, including those on hemodialysis, was similar between the intervention and the control groups (RR 1.15; 95% CI 0.90, 1.45; *p* = 0.26), but the risk of minor bleeding events was substantially higher in the intervention group (RR 1.57; 95% CI 1.18, 2.10; *p* = 0.002) (Figure 4a,b).

#### Subgroup Analysis in Bleeding Risks Associated with the Extent of Renal Insufficiency

A subgroup analysis revealed that the risk of both major and minor bleeding was substantially elevated with the intervention in patients with severe CKD (eGFR < 30 mL/min) or on hemodialysis (RR 1.30; 95% CI 1.09, 1.55; *p* = 0.002), but not in CKD patients with moderate renal insufficiency eGFR (RR 1.22; 95% CI 0.98, 1.53; *p* = 0.08) (Figure 4c).

### 3.5. Intermediate Outcomes in Patients with CKD

The intermediate outcome, expressed as a measurement of platelet function, was assessed in six studies [5,31,32,33,34,35], two [5,31] in patients with CKD and four [32,33,34,35] in patients on hemodialysis (Table 2). Platelet activity was significantly reduced with DAPT with prasugrel or ticagrelor and triple therapy, even with a reduced dose of prasugrel and ticagrelor. Nevertheless, doubled clopidogrel LD or MD failed to decrease platelet reactivity.

## 4. Discussion

In this study, we investigated the clinical efficacy and safety of different antiplatelet therapy regimens with enhanced platelet-inhibition activity over a standard dose of clopidogrel-based DAPT in subjects with CKD. We found that the interventions, antiplatlet therapy regimens with enhanced antiplatelet activity over standard clopdigorel-based DAPT, substantially improved clinical outcomes, including all-cause mortality (RR 0.67, *p* = 0.003), MACE (RR 0.79, *p* < 0.00001), and MI (RR 0.28, *p* = 0.0007) without major bleeding (RR 1.14, *p* = 0.33) in patients with ACS and CKD. Also, the risk of bleeding was substantially greater in patients with severe CKD (eGFR < 30 mL/min) than those with moderate renal insufficiency. Platelet activity is significantly inhibited with prasugrel, ticagrelor, and triple therapy, but not with double-dose clopidogrel in patients with CKD, including those on hemodialysis.

The current guidelines highly recommend DAPT as a part of ACS management to improve patient outcomes and prognosis, as platelet aggregation plays a pivotal role in ACS progression. However, the appropriate antiplatelet therapy regimen in those with CKD remains ambiguous [1,2]. According to an observational study, the majority of people with CKD are discharged with a standard dose of clopidogrel-based regimen with LD of 300 mg and MD of 75 mg in addition to low-dose aspirin. The percentage of patients discharged on this regimen increases according to the degree of renal dysfunction [16]. However, people with CKD have complex hemostatic properties that may hinder the clinical benefits of ACS management. This is implied by an elevated ischemic risk despite proper antiplatelet therapy and consequent increasing mortality [5,8].

Previous studies have reported a 6–12% intraindividual variability of the clopidogrel response secondary to multiple factors, including genetic polymorphism of cytochrome (CYP) 2C19, as clopidogrel is mainly metabolized into its active ingredient by CYP 2C19. [36,37,38]. However, the underlying mechanism of clopidogrel-response variability in CKD patients including those on dialysis remains unclear, as the degree of reduced clopidogrel response increases with renal insufficiency, and studies report patients on dialysis have poor responses to clopidogrel regardless of having the 2C19*2 alleles, the loss-of-function genotype [9,14]. Considering the marked increased risk of ischemic events and mortality in patients with CKD, and the fact that a substantial number of the ACS populations has CKD as an underlying comorbidity, the clinical outcomes of antiplatelet therapy should be clarified to determine a suitable DAPT regimen for these populations [8].

We evaluated the efficacy of intervention composed of five different antiplatelet regimens with enhanced platelet inhibition activity to overcome clopidogrel-response variability in CKD patients: doubled LD of clopidogrel, doubled MD of clopidogrel, prasugrel, ticagrelor-based DAPT, and triple antiplatelet therapy with cilostazol. Although the intervention provided considerable clinical benefits over a standard dose of clopidogrel-based DAPT, each intervention must be assessed before clinical application in CKD patients. The clopidogrel dose has been doubled in patients with diminished clopidogrel response, but our results indicate that the clinical benefits of this are relatively insignificant. Furthermore, the clinical benefits of triple therapy with cilostazol, a phosphodiesterase III inhibitor, must also be clarified because mortality was the only improved clinical outcome, despite the addition of medication. Similar to the result of previous studies, this study revealed that replacing clopidogrel with a potent oral P2Y12 inhibitor such as prasugrel or ticagrelor provides beneficial clinical outcomes in CKD patients, as implied by a substantially lowered all-cause mortality risk, MACE, and MI without elevated bleeding risks [15].

We also found that the interventions did not increase the risk of major bleeding in those with CKD despite enhanced antiplatelet activity, but the risk of bleeding was markedly increased in patients with CKD and renal function severely decreased, including those on hemodialysis. Our analysis indicated that renal insufficiency may play a crucial role in increasing bleeding risks. An observational study revealed the most substantial incidence of bleeding events occur in those with stage 4 CKD [16]. Additionally, a large proportion of stage 4 CKD patients discharged with potent P2Y12 inhibitors switched from a DAPT regimen to a clopidogrel-based regimen secondary to bleeding events. Considering the risks and benefits of antiplatelet use, a standard dose of clopidogrel-based DAPT can be preferred in those with severe CKD, including those on dialysis. Further research needs to elucidate a clear mechanism of bleeding associated with renal dysfunction and an approach to attenuate bleeding risks from potent P2Y12 inhibitors.

Recently, a real-world observational study comparing clinical efficacy and bleeding risk between clopidogrel and ticagrelor in patients with a risk of high bleeding, including those with renal insufficiency, demonstrated substantially reduced risk of net adverse clinical endpoints (NACE), composites of all-cause death, MI, stroke, and major bleeding events at one year, and MACE in patients treated with ticagrelor when compared to those with clopidogrel [39]. Nonetheless, we cannot affirm that ticagrelor is a drug of choice over other regimens in patients with mild to moderate renal insufficiency at this point because the authors suggested DAPT duration is associated with the risk of ischemic and bleeding events at one year after MI based on the results of no difference in the risk of NACE and MACE between ticagrelor and clopidogrel treated patients after adjusting factors increasing bleeding risk such as age, PCI, smoking status, creatinine clearance, previous MI and number of high-risk bleeding criteria [39]. Another study, however, demonstrated controversial results; prolonged use of low dose ticagrelor (60 mg twice daily) after 12 months of DAPT provided long-term efficacy without major bleeding events [40]. Summing up, DAPT with potent P2Y12 inhibitors may be a plausible treatment option for CKD patients, but further controlled studies on the impact of duration and type of DAPT, along with disease-related factors on ischemic and bleeding outcomes, in CKD patients are warranted. This is because CKD patients possess multiple underlying factors that may subsequently affect hemodynamics and ischemic/bleeding risk, and the clear mechanism of these effects are yet to be determined.

This is the first meta-analysis investigating five different and potentially appropriate antiplatelet therapy regimens with enhanced antiplatelet activity over standard clopidogrel-based DAPT in patients with CKD. We assessed the clinical safety of different antiplatelet therapy regimens in patients on hemodialysis. Based on our study results, patients with moderate renal insufficiency may clinically benefit from the antiplatelet therapy regimen with enhanced antiplatelet activity (intervention) without bleeding risks. However, uncertainty remains regarding the use of alternative antiplatelet therapy regimens in patients on hemodialysis secondary to the most significant bleeding risk and unavailable clinical outcomes, such as mortality and MACE. Although the current guidelines do not recommend adjusting the dose of P2Y12 inhibitors, dosage adjustment should be considered in these patient populations based on the results which displayed significantly lowered platelet function with low doses of prasugrel and ticagrelor when compared to a standard and doubled clopidogrel dose [1,2,31,33,41]. Additionally, a PEGASUS-TIMI trial has revealed that ticagrelor treatment of 60 mg twice daily in patients with high-risk features such as old age, diabetes mellitus requiring medication, or renal insufficiency (eGFR < 60 mL/min) provided similar cardiovascular protection with better safety profiles when compared to ticagrelor 90 mg twice daily [42]. Nonetheless, treatment with ticagrelor 60 mg is recommended in patients with extended DAPT beyond 12 months, implying validated clinical efficacy of low-dose ticagrelor-based DAPT for ACS management in CKD is limited [40]. Hence, cautious selection of antiplatelet therapy regimens with specific monitoring plans are encouraged, and further studies assessing clinical outcomes with reduced doses of prasugrel or ticagrelor should be considered in CKD patients.

This study has some limitations. The included studies had different study designs and outcome measurements, which may hinder their clinical application. Moreover, fewer patients with CKD received the intervention than a standard dose of clopidogrel-based DAPT. Also, a small number of studies evaluated each intervention, which may attenuate the validity of these study results. These issues may be justified because CKD patients are a vulnerable population that is under-represented in RCTs. This accentuates the importance of this study as it provides insight on potential antiplatelet therapies in clopidogrel-resistant patients with CKD who have been diagnosed with ACS. As CKD patients are at considerable risk for ischemic and bleeding events, further research that determines personalized factors associated with the beneficial clinical outcomes of antiplatelet therapy is needed. This will help provide appropriate antiplatelet therapy in clopidogrel-resistant CKD patients without increasing bleeding events.

## 5. Conclusions

In conclusion, the interventions composed of five different antiplatelet activities with enhanced antiplatelet activities over standard clopidogrel-based DAPT considerably enhanced clinical outcomes regarding all-cause mortality, MACE, and MI without bleeding risks in CKD patients diagnosed with ACS. This suggests that the interventions are plausible treatment alternatives in CKD patients who may have reduced clopidogrel response. However, standard doses of clopidogrel-based DAPT is recommended in those with severe CKD, including those on hemodialysis, based on the studies available with unclear clinical outcomes and increased bleeding risk. Further studies investigating personalized factors associated with improved clinical outcomes are warranted to implement the most appropriate antiplatelet therapy in this population.

## Figures and Tables

**Table 1:** PICOS of this study.

Component	Definition
P (patients)	Patients in ACS with CKD, including HD
I (intervention)	DAPT regimen with enhanced antiplatelet activity (or reduced platelet reactivity) to overcome response variability of clopidogrel and is composed with [13]: (1) Doubled LD of clopidogrel-based DAPT (2) Doubled MD of clopidogrel-based DAPT (3) Ticagrelor-based DAPT (4) Prasugrel-based DAPT, and (5) Triple antiplatelet therapy with cilostazol
C (comparator)	Standard dose of clopidogrel-based DAPT
O (outcomes)	Efficacy outcomes: all-cause or cardiac-related mortality, MACE, MI, stent thrombosis; safety outcomes: major or minor bleeding; intermediate outcomes: IPA and PRU
S (study design)	RCTs, observational studies, and prospective studies

Abbreviations. ACS: acute coronary syndrome, CKD: chronic kidney disease, DAPT: dual antiplatelet therapy, HD: hemodialysis, IPA: inhibition of platelet aggregation, LD: loading dose, MD: maintenance dose, PRU: platelet reactivity unit, RCT: randomized clinical trial.

**Table 2:** Study characteristics.

Author Name	Study Region	Study Design	Patient Population	Intervention	Comparator (Control)	Duration	Efficacy	Safety
**CKD Populations (eGFR < 60 mL/min)**								
Barber et al. (2017) [25]	U.S.	Multicenter, observational study	ACS patients with CKD undergoing PCI (*n* = 5613)	Prasugrel + ASA (*n* = 617)	Clopidogrel +ASA (*n* = 4996)	1 year	No significant difference in MACE, death, and MI	No significant difference in bleeding
Choi et al. (2012) [26]	Korea	Prospective, multicenter, online registry of Korea (KAMIR)	AMI patients with renal dysfunction (*n* = 2288)	Triple therapy (ASA+clopidogrel+cilostazol) (*n* = 701)	Clopidogrel +ASA (*n* = 1587)	Not available	Significantly lower rates of in-hospital death (6.7% vs. 11.3%, *p* = 0.001) and 1-month MACE (11.1% vs. 16.3% *p* = 0.002) in triple therapy group but no difference in 12-month MACE	No significant difference in bleeding, in-hospital major bleeding (*p* = 0.870)
Edfors et al. (2018) [27]	Sweden	Swedish Web-System for Enhancement and Development of Evidence-Based Care in Heart Disease Evaluated According to Recommended Therapies (SWEDEHEART) registry study	NSTEMI and STE15MI patients discharged with DAPT and eGFR less than 60 mL/min (*n* = 11,538)	Ticagrelor 180 mg LD followed by 90 mg BID +ASA (*n* = 2196)	Clopidogrel + ASA (*n* = 9342)	12 months	Ticagrelor as compared with clopidogrel was associated with a lower 1-year risk of the composite outcome (eGFR 30–60: 0.82 (0.70 to 0.97), eGFR < 30: 0.95 (0.69 to 1.29), *p = 0.55*)across the eGFR strata.	Ticagrelor as compared with clopidogrel was associated with a higher risk of bleeding (eGFR 30–60: 1.13 (0.84 to 1.51), eGFR < 30: 1.79 (1.00 to 3.21), *p* for interaction = 0.30) across the eGFR strata.
James et al. (2010) [28]	Multinational	Post hoc analysis of a multicenter, randomized, double-blind trial	ACS patients with chronic kidney disease (*n* = 3237)	Ticagrelor 180 mg LD followed by 90 mg BID+ Aspirin (75–100 mg)	Clopidogrel 300 mg LD followed by 75 mg daily + Aspirin (75–100 mg)	12 months	Significantly reduced primary end points (CV death, MI, stroke) of HR 0.77 [0.65–0.9], *p* = 0.03 and mortality of HR 0.72 [0.58–0.89], *p* = 0.02 in ticagrelor	No significant difference in major bleeding rates (HR: 1.07 [0.88–1.30]), fatal bleedings (HR: 0.48 [0.15–1.54]), and non-CABG-related major bleedings (HR:1.29 [0.97–1.68])
Kim et al. (2012) [29]	Korea	Prospective, open, observational, multicenter on-line registry of Korea (KAMIR)	STEMI patients undergoing PCI with CKD within 24 h of onset (*n* = 1457)	Clopidogrel 600 mg LD+75 mg MD + ASA 100mg	Clopidogrel 300mg LD+ 75mg MD + ASA 100mg	Not available	No difference in MACE at 1 month (15.6 vs. 16.4%, *p* = 0.700) and 12 months (19.0% vs. 21.3%, *p* = 0.32)	In-hospital major bleeding rate was similar (0.8% vs. 0.2%, *p* = 0.09).
Liang et al. (2015) [30]	China	Prospective, randomized, open-label, parallel-group, single-center study	CAD patients with CKD undergoing PCI with DES (*n* = 370)	Clopidogrel 300 mg LD followed by 150 mg daily + ASA 100 mg daily (*n* = 186)	Clopidogrel 300 mg LD followed by 75 mg daily + ASA 100mg daily (*n* = 184)	30 days	Significantly lower rates of stent thrombosis (1.1% vs. 4.9%, *p* = 0.03) and MACE (2.7% vs. 7.6%, *p* = 0.03) in patients who received 150 mg	No significant difference in major(1.6% vs. 1.1%, *p* = 1.00) or minor (5.4% vs. 2.2%, *p* = 0.11) bleeding
Melloni et al. (2015) [5]	Multinational	Phase 3, randomized, double-blinded, double-dummy, active-control study	ACS patients enrolled in TRIOLOGY-ACS study (*n* = 8953)	Prasugrel 30 mg LD followed by 10 mg daily (5 mg daily for patients older than 75 years or if < 60 kg +ASA	Clopidogrel 300 mg LD followed by 75 mg daily+ ASA	30 months	Substantially reduced PRU in prasugrel in all three CKD stages, Difference in PRU between (prasugrel-clopidogrel): severe CKD: −81.8 [−130.6, −33.1] moderate CKD: −70.8 [−84.8, −56.8) normal/mild CKD −101.4 [−110.1, −92.7]	Not available
Nishi et al. (2017) [31]	Japan	Post hoc analysis of a single-center, prospective, crossover study	Japanese patients undergoing PCI (*n* = 53 total, *n* = 15 for CKD and *n* = 38 for non-CKD)	Prasugrel 3.75 mg daily + ASA 100 mg	Clopidogrel 300 mg LD followed by 75 mg daily+ ASA 100 mg	28 days (crossover at day 14)	Significantly lower PRU in prasugrel treated patients (165.3 ± 61.8 vs. 224.3 ± 57.0, *p* = 0.002)	Not available
***Patients on HD***								
Jeong et al. (2015) [32]	Korea	Single-center, prospective, randomized, crossover study	Patients with kidney failure with HTPR on HD (*n* = 25)	Ticagrelor 180 mg LD followed by 90 mg BID +ASA 100 mg	Clopidogrel 300 mg LD followed by 75 mg daily + ASA 100 mg	10 weeks	More rapid and greater platelet inhibition in ticagrelor treated group (*p* < 0.05)	Two clinically relevant cases of minor bleeding in ticagrelor-treated group (1 arteriovenousfistula bleeding and 1 oral bleeding)
Kim et al. (2017) [33]	Korea	Prospective, randomized, single-center study	ESRD patients on regular HD (*n* = 52)	(1) Ticagrelor 90 mg LD followed by 90 mg daily +ASA (*n* = 13) OR (2) Ticagrelor 180 mg LD followed by 90 mg BID+ ASA(*n* = 21)/	Clopidogrel 300 mg LD followed by 75 mg daily +ASA(*n* = 18)	14 days	Significant difference in IPA in low ticagrelor group compared to clopidogrel treated group. Standard ticagrelor group showed the highest IPA (ANCOVA < 0.001)	No bleeding events in low-dose ticagrelor BARC Type I (gum bleeding) events: clopidogrel (5.9%) standard ticagrelor (5.6%) BARC Type 2 events (arteriovenous fistula bleeding) standard ticagrelor (5.6%)
Park et al. (2009) [34]	Korea	Prospective, randomized, open-label single-center study	Patients with CRF (75% patients on HD) (*n* = 36)	Clopidogrel 600 mg LD followed by 150 mg daily +ASA 100 mg	Clopidogrel 300 mg LD followed by 75 mg daily + ASA 100 mg	4 weeks/30 days	No significant difference in PRU (302 ± 81 vs. 308 ± 70, *p* = 0.824) and mean percentage of inhibition (23.4 ± 14.4 vs. 21.3 ± 16.0, *p* = 0.808)	Gastrointestinal ulcer bleeding in 1 patient who received clopidogrel at 150 mg
Woo et al. (2011) [35]	Korea	Prospective, open, randomized platelet function study	CKD patients undergoing HD who received PCI (*n* = 74)	(1) Clopidogrel 150 mg/day +ASA 100 mg (*n* = 25) (2) Triple therapy (clopidogrel 75 mg + cilostazol+ ASA 100 mg) (*n* = 25)	Clopidogrel 75 mg + ASA 100 mg (*n* = 24)	14 days	The rate of high on-treatment platelet activity was significantly lower in triple therapy (10% vs. 43% vs. 32% *p* < 0.05)	Gastrointestinal ulcer bleeding in 1 patient who received clopidogrel at 150 mg

## Supplementary Materials

The following are available online at https://www.mdpi.com/2075-4426/11/3/222/s1, Figure S1: Funnel plots of (a) all-cause mortality; and (b) major bleeding, Table S1: Quality assessment of randomized controlled trials, Table S2: Quality assessment of non-randomized controlled trials.
