# Supplementary material for: P2Y12 Antiplatelet Choice for Patients with Chronic Kidney Disease and Acute Coronary Syndrome: A Systematic Review and Meta-Analysis
Source: J Pers Med. 2021 Mar 21;11(3):222. doi: 10.3390/jpm11030222 (PMC8004167; doi:10.3390/jpm11030222)

Table S1. Quality assessment of randomized controlled trials

	Random sequence generation	Allocation concealment	Blinding of participants and personnel	Blinding outcome assessment	Incomplete outcome data	Selective reporting	Other bias
James et al. (2010) [22]	L	L	L	L	U	L	L
Jeong et al. (2015) [26]	L	L	L	U	U	L	U
Kim et al. (2017) [27]	L	L	H	U	U	L	U
Liang et al. (2015) [24]	U	H	H	U	U	U	L
Melloni et al. (2015) [7]	L	L	L	L	L	L	L
Park et al. (2009) [28]	U	H	H	H	U	L	U
Woo et al. (2011) [29]	L	H	H	H	U	L	L

Table S2. Quality assessment of non-randomized controlled trials

	Selection of participants	Confounding variables	Measurement of intervention	Blinding for outcome assessment	Incomplete outcome data	Selective outcome reporting
Barber et al. (2017) [19]	L	L	L	H	U	L
Choi et al. (2012) [20]	L	U	L	H	L	L
Edfors et al. (2018) [21]	L	L		H	U	L
Kim et al. (2012) [23]	L	L	L	H	U	U
Nish et al. (2017) [25]	H	U	U	H	U	L

Figure S1. Funnel plots of (a) all-cause mortality; and (b) major bleeding

(a)

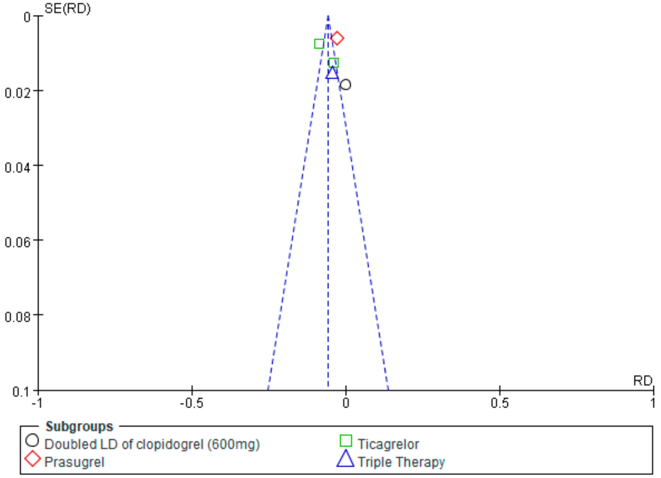

(b)

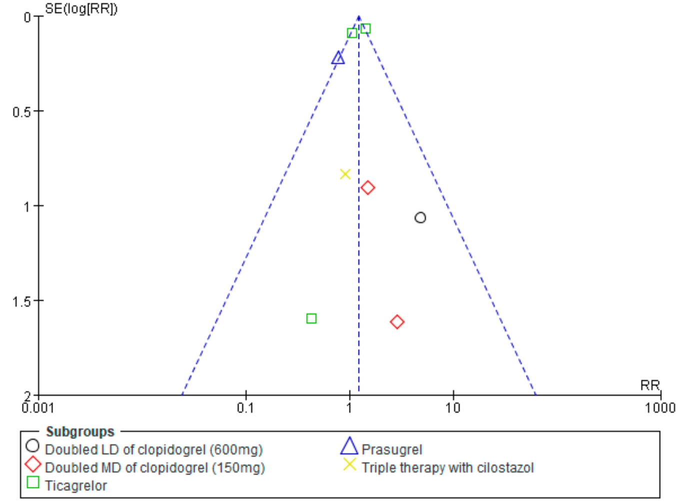

Supplement: Supplementary file 1 [file jpm-11-00222-s001.pdf]
